# Transcriptional coupling and repair of 8-OxoG activate a RecA-dependent checkpoint that controls the onset of sporulation in *Bacillus subtilis*

**DOI:** 10.1038/s41598-021-82247-8

**Published:** 2021-01-28

**Authors:** Valeria P. Suárez, Lissett E. Martínez, Hilda C. Leyva-Sánchez, Luz I. Valenzuela-García, Reyna Lara-Martínez, Luis F. Jiménez-García, Norma Ramírez-Ramírez, Armando Obregon-Herrera, Mayra Cuéllar-Cruz, Eduardo A. Robleto, Mario Pedraza-Reyes

**Affiliations:** 1 Division of Natural and Exact Sciences, Department of Biology, University of Guanajuato, Guanajuato, Mexico; 2 School of Life Sciences, University of Nevada, Las Vegas, NV USA; 3 Department of Cell Biology, Faculty of Sciences, National Autonomous University of Mexico (UNAM), Circuito Exterior, Ciudad Universitaria, Cd. Mx., Coyoacán, 04510 Mexico City, Mexico

**Keywords:** Genetics, Microbiology, Molecular biology

## Abstract

During sporulation *Bacillus subtilis* Mfd couples transcription to nucleotide excision repair (NER) to eliminate DNA distorting lesions. Here, we report a significant decline in sporulation following Mfd disruption, which was manifested in the absence of external DNA-damage suggesting that spontaneous lesions activate the function of Mfd for an efficient sporogenesis. Accordingly, a dramatic decline in sporulation efficiency took place in a *B. subtilis* strain lacking Mfd and the repair/prevention guanine oxidized (GO) system (hereafter, the ∆GO system), composed by YtkD, MutM and MutY. Furthermore, the simultaneous absence of Mfd and the GO system, (i) sensitized sporulating cells to H_2_O_2_, and (ii) elicited spontaneous and oxygen radical-induced rifampin-resistance (Rif^r^) mutagenesis. Epifluorescence (EF), confocal and transmission electron (TEM) microscopy analyses, showed a decreased ability of ∆GO ∆*mfd* strain to sporulate and to develop the typical morphologies of sporulating cells. Remarkably, disruption of *sda*, *sirA* and *disA* partially, restored the sporulation efficiency of the strain deficient for Mfd and the ∆GO system; complete restoration occurred in the RecA^−^ background. Overall, our results unveil a novel Mfd mechanism of transcription-coupled-repair (TCR) elicited by 8-OxoG which converges in the activation of a RecA-dependent checkpoint event that control the onset of sporulation in *B. subtilis*.

## Introduction

When conditions are no longer appropriate for growth, a subpopulation of stationary-phase *B. subtilis* cells triggers a developmental pathway leading to the synthesis of highly resistant and differentiated cells, termed spores^1,2^. During the initial stages of this developmental process, the sporulating cells experience an asymmetric cell division that generates two compartments of different size, the mother cell (larger compartment) and the forespore (smaller compartment)^3^. During the early sporulation stages the chromosome exhibits a final round of replication to ensure the existence of two chromosomal copies during the asymmetric division. Upon establishment of the sporulation septum one of these chromosomal copies segregates to the forespore compartment^1,3^. From this step forward, sporogenesis is orchestrated by a spatio-temporal program of gene expression taking place in both cross talking compartments^1,3–5^. It has been proposed that the damages inflicted to the sporangia’s chromosomes must be immediately corrected to prevent deficiencies in this developmental program and insure a proper sporulation process^6–8^. Previous observations have revealed that sporulating cells deploy repair and tolerance mechanisms to safeguard the integrity of the sporangia’s chromosomes^9–13^. Accordingly, in sporangia, helix-distorting DNA lesions inflicted by ultraviolet C light and mitomycin C are mainly processed by the transcriptional coupling repair (TCR) factor Mfd and the nucleotide excision repair pathway (NER)^13,14^. Furthermore, a recent report revealed that the SOS response is active during sporulation and that RecA, is required to counteract genetic lesions inflicted by physical and alkylating factors^9^. However, two additional sporulation roles have been attributed to RecA, firstly as a factor that regulates the levels of phosphorylated Spo0A during the onset of sporulation, and secondly blocking replication and vegetative growth in further stages of this developmental pathway^15–17^. As noted above, Mfd and the NER system counteract the cytotoxic and genotoxic effects promoted by physical and chemical factors that promote bulky DNA lesions^13,18–20^. However, in the absence of external DNA damaging factors, the sole absence of Mfd affected sporulation in *B. subtilis*^13^, suggesting that spontaneous DNA lesions in actively transcribed genes require Mfd for a proper sporogenesis. In support of this concept, here we report that disabling of the repair/prevention guanine oxidized (GO system) in a Mfd-deficient genetic background induced a marked decrease in *B. subtilis* sporulation. Results from TEM and confocal microscopies demonstrated the sporulation defect, including, the incapability of the ∆*mfd* ∆GO strain to generate typical sporangia and mature spores. Furthermore, in reference to WT sporulating cells, the ∆GO ∆*mfd* mutant was severely affected by the ROS-promoter agent H_2_O_2_ and exacerbated its spontaneous and oxygen radical-induced mutagenesis. Interestingly, disruption of *recA*, *sda, disA* or *sirA* suppressed the sporulation deffect exhibited by the YtkD/MutM/MutY/Mfd-deficient strain. Overall our results support the notion that transcriptional coupling of 8-OxoG repair activates checkpoint events dependent on RecA, Sda, SirA and DisA with a direct impact on the onset and progression of *B. subtilis* sporulation.

## Results

### Mfd and the GO system are required for an efficient sporulation process in *B. subtilis*

**Figure 1:**
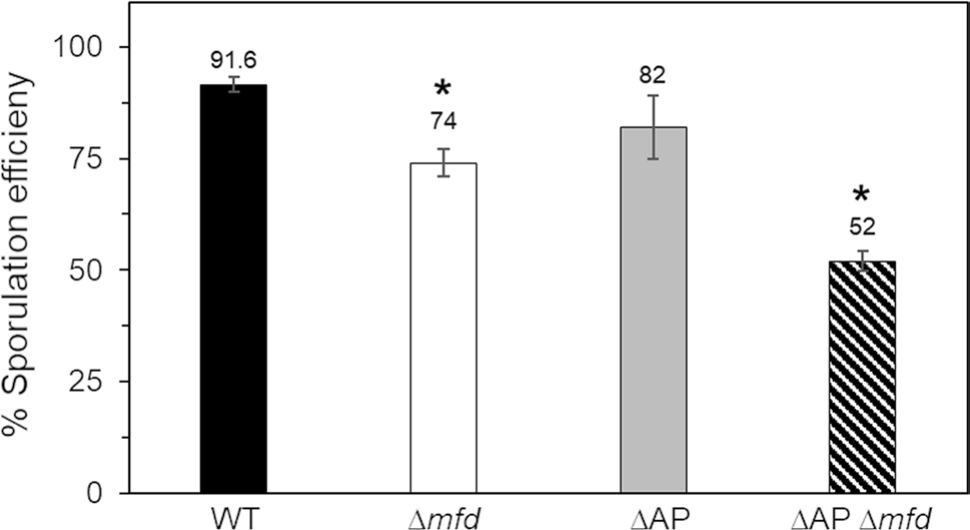
Sporulation efficiency of strain 168 derivatives. The strains were induced to sporulate in DSM for 24 h at 37 °C and assessed for sporulation efficiency by the heat-killing method. Asterisks indicate statistically significant differences between strains as determined by one-way analysis of variance (ANOVA) followed by a Tukey’s post-hoc test; *P* < 0.05. Values are expressed as mean values ± standard deviation of at least three independent experiments.

**Figure 2:**
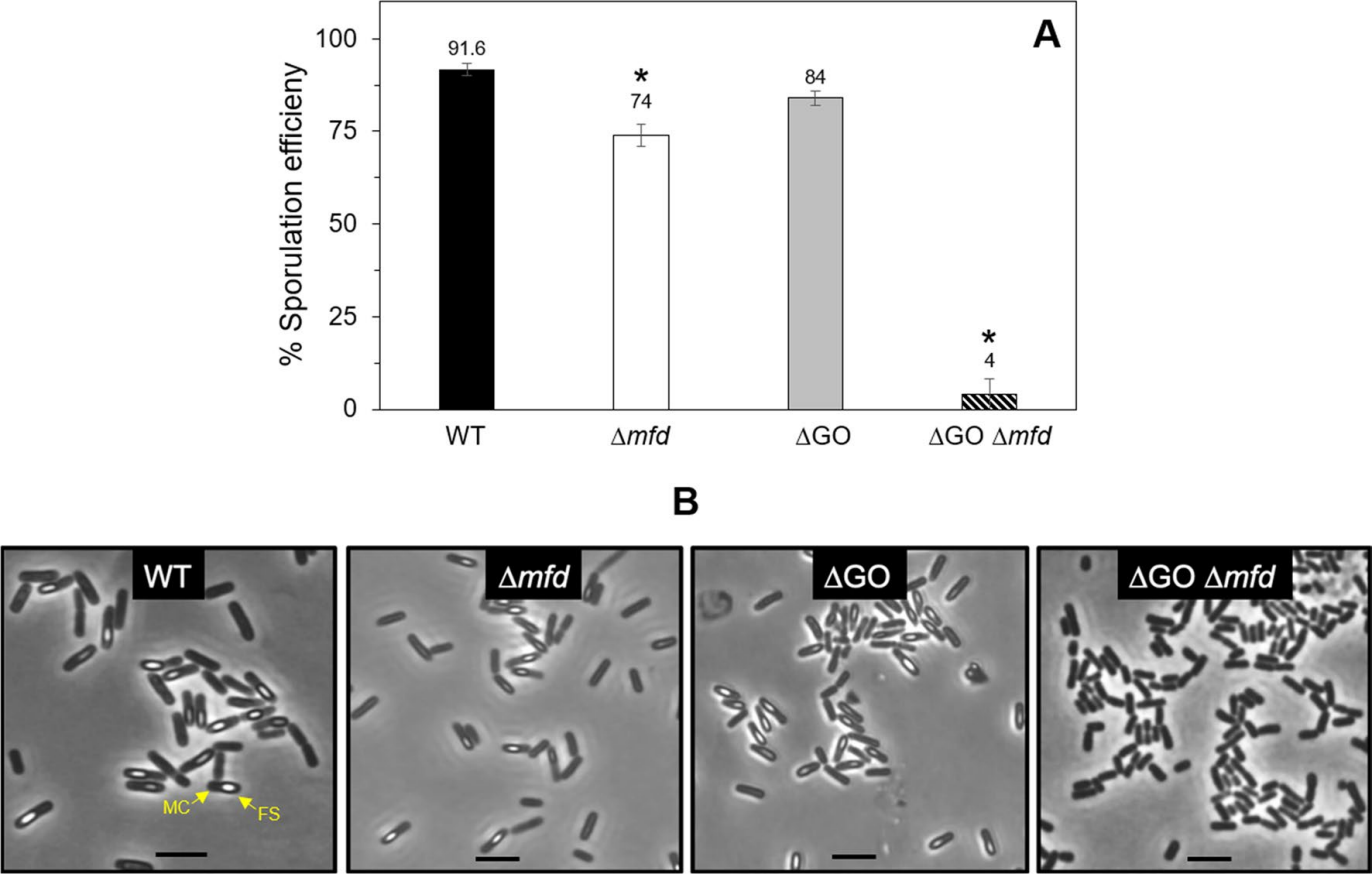
(**A**) Sporulation efficiency of strain 168 derivatives. The strains were induced to sporulate in DSM for 24 h at 37 °C and assessed for sporulation efficiency by the heat-killing method. Asterisks indicate statistically significant differences between strains as determined by one-way analysis of variance (ANOVA) followed by a Tukey’s post-hoc test; P < 0.05. Values are expressed as mean values ± standard deviation of at least three independent experiments. (**B**) Strains indicated were induced to sporulate at 37 °C, and 9 h after t_0_, cell samples were collected and analyzed by confocal microscopy as described in “Materials and methods” section. MC and FS, mother cell and forespore compartments. The scale bar is 2 μm.

A previous report revealed that inactivation of *mfd* affected the sporulation process of *B. subtilis*, even in the absence of external genotoxic factors^13^. To explore whether spontaneous DNA lesions elicited by ROS are involved in this sporulation defect, the AP-endonucleases Nfo, ExoA, Nth or the full GO system^21^ were disabled in a *B. subtilis mfd* knockout, and the resulting strains were tested for sporulation efficiency. While no obvious effects were observed in the ∆AP or ∆GO strains; the absence of Mfd resulted in a significant decline in sporulation in comparison with the WT parental strain (Fig. 1). Strikingly, while *mfd* disruption in the ∆AP strain decreased ~ 40% the sporulation efficiency (Fig. 1); the developmental sporulation process was almost completely abolished following disruption of Mfd in the ∆GO strain (Fig. 2A).

Confocal phase-contrast microscopy analysis of cell samples collected during the stage t_9_ of the sporulation process corroborated that while the WT, ∆*mfd* and ∆GO strains generated typical sporangia with mature refringent spores, in this stage, the ∆*mfd* ∆GO strain was incapable of generating cells with a typical sporulating phenotype (Fig. 2B). Altogether, these results strongly suggest that AP sites and 8-OxoG lesions that compromise sporulation require a functional Mfd protein.

### The GO system together with Mfd contributes to an efficient *B. subtilis* sporulation process

**Figure 3:**
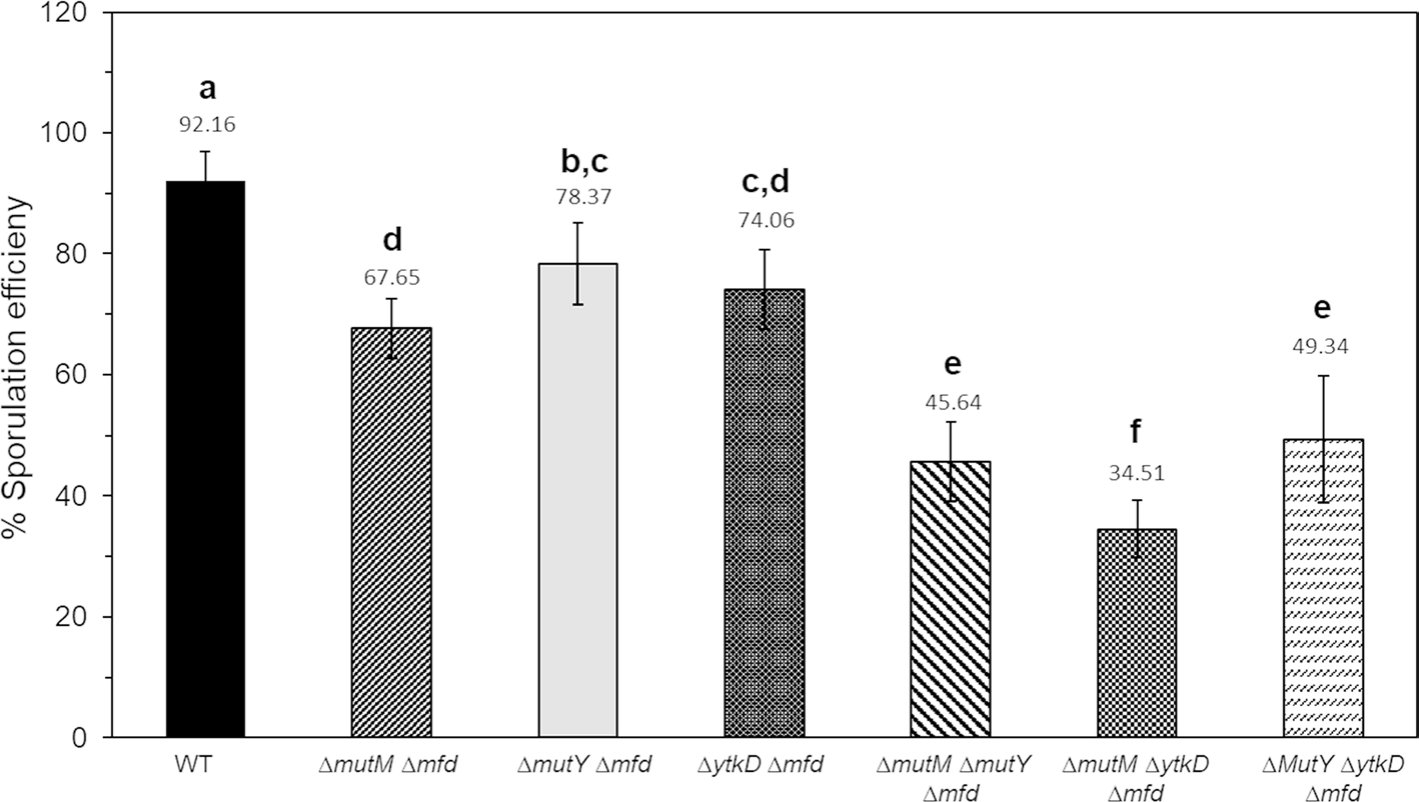
Sporulation efficiency of strain 168 derivatives. The strains were induced to sporulate in DSM for 24 h at 37 °C and assessed for sporulation efficiency by the heat-killing method. Letters a, b, c and d, indicate significant differences between strains as determined by one-way analysis of variance (ANOVA) followed by a Tukey’s post-hoc test; *P* < 0.05. Values are expressed as mean values ± standard deviation of at least three independent experiments.

The strong sporulation defect detected in the ∆*mfd* ∆GO strain was of interest and further analyzed. The simultaneous action of the three components of the GO system counteract the mutagenic and cytotoxic effects of 8-OxoG; however, they accomplish this task through different mechanisms. Whereas MutM specifically hydrolyzes 8-OxoG from DNA, MutY catalyzes the elimination of adenine incorrectly paired with oxidized guanine^22–24^. In contrast, following hydrolysis of 8-Oxo-dGTP, the nucleotide diphosphohydrolase YtkD, avoids the incorporation of 8-OxoG to replicating DNA^25,26^. Therefore, we disabled one or two genes encoding functions of the GO system in the Mfd-deficient strain to better measure their independent and combined contributions to the sporulation process of this strain. Our results revealed similar sporulation efficiencies among the *mfd* strain and its derived strains which carried single disruptions on *mutM*, *mutY* or *ytkD* (Figs. 2, 3). However, a significant decline in sporulation was observed upon disruption of two gene encoding components of the GO system in the ∆*mfd* strains. The simultaneous disruption of *mutM*/*mutY*, *mutY*/*ytkD* or *mutM*/*ytkD* decreased the sporulation efficiency of the strain lacking Mfd between 40 and 50% (Figs. 2, 3). Overall, these results and those shown in Fig. 2, indicate that disruption of the three components of the GO system is necessary to induce a marked decrease in the sporogenesis of the Mfd-deficient strain.

### The GO system and Mfd confers protection to sporulating cells from ROS-promoted DNA damage and mutagenesis

**Figure 4:**
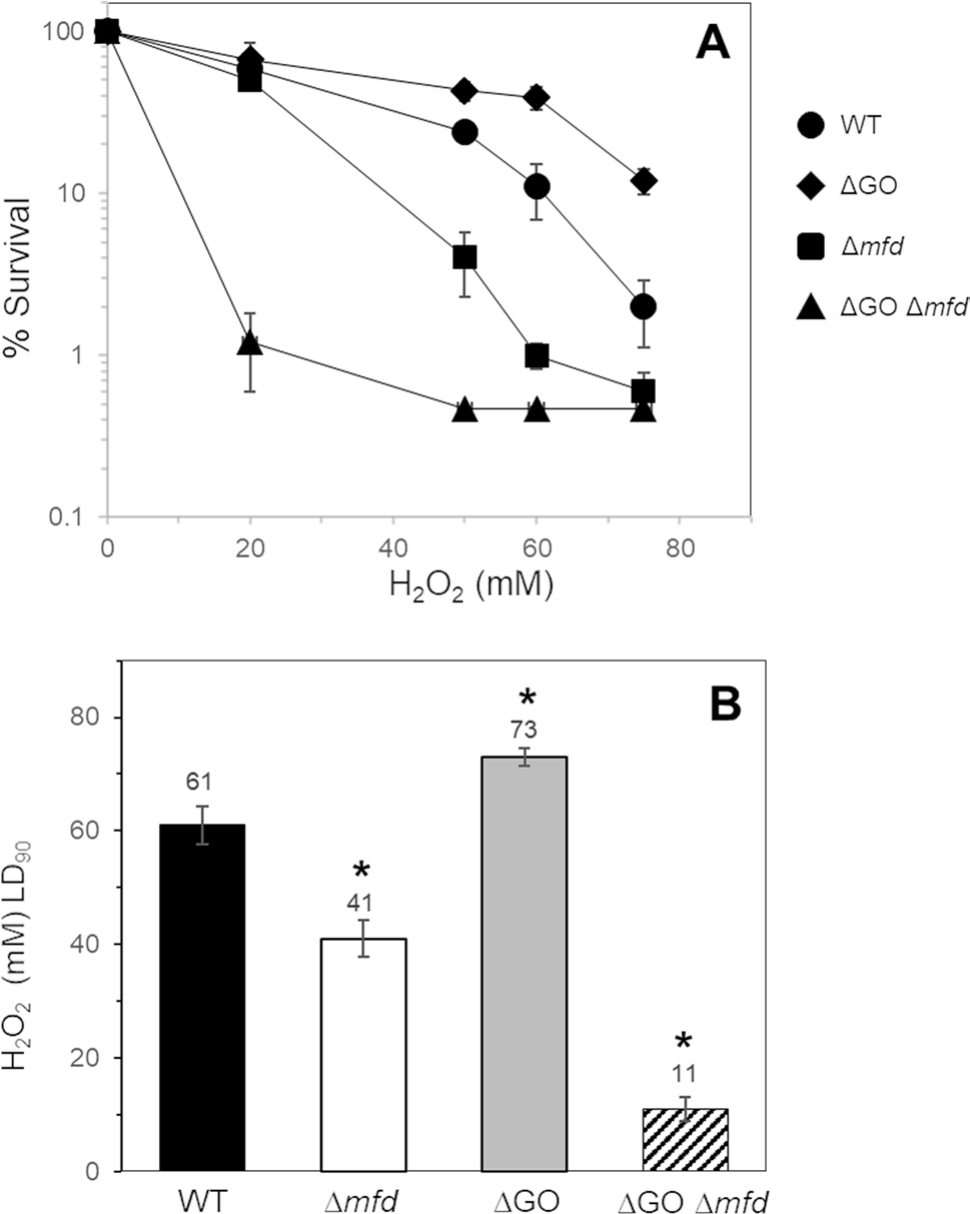
(**A**) Resistance to H_2_O_2_ of strain 168 derivatives during sporulation. Sporulating cells of strains *B. subtilis* wild type (WT; filled circle), ∆GO (filled diamond), ∆*mfd* (filled square) and ∆GO ∆*mfd* (filled triangle) were treated with increasing doses of H_2_O_2_ at 4.5 h after the onset of sporulation, and cell survival was determined as described in Materials and Methods. The LD_90_ values (**B**) were calculated for each strain from the dose–response graphs (**A**). Data are expressed as the average ± SD of at least three independent experiments. Asterisks indicate statistically significant differences between strains as determined by one-way analysis of variance (ANOVA) followed by a Tukey’s tests; *P* < 0.05.

**Figure 5:**
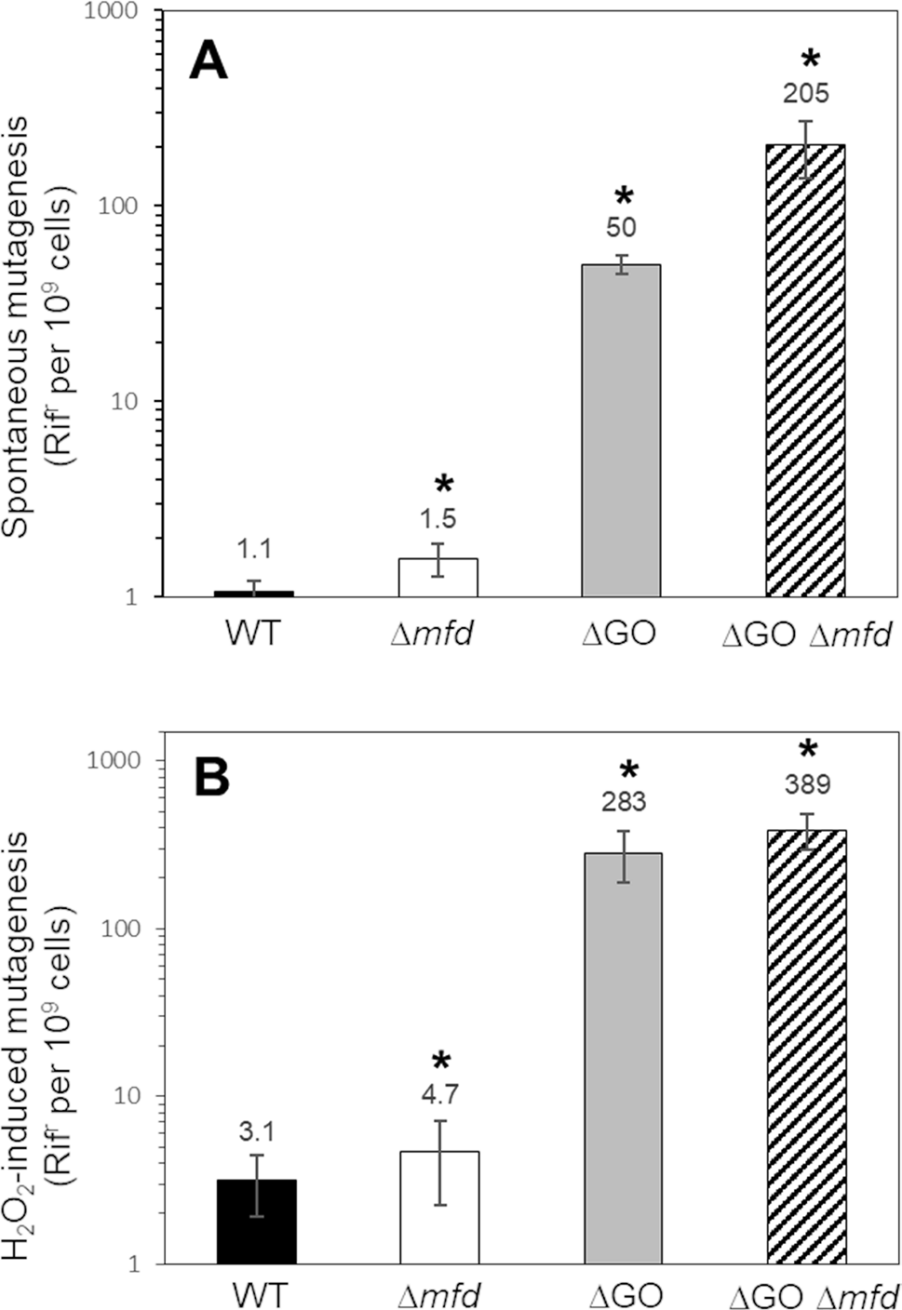
Frequencies of mutation to Rif^r^. Cells of *B. subtilis* wild type (WT; black bars), ∆GO (gray bars), ∆*mfd* (white bars) and ∆GO ∆*mfd* (hatched bars) strains, were sporulated in liquid DSM and left untreated (**A**) or treated (**B**) with the LD_50_ for each strain of H_2_O_2_ (**B**) at 4.5 h after the onset of sporulation. The levels of Rif^r^ cells with (**B**) or without (**A**) treatment exposure were determined. Each bar represents the the average ± SD of at least three independent experiments. Asterisks indicate statistically significant differences between strains as determined by U Mann–Whitney test; *P* < 0.05.

The TCR factor Mfd and the prevention/repair GO system are required for *B. subtilis* sporogenesis (Fig. 1). Therefore, we determined the effects of single and combined deficiencies of both functions on the protection of sporulating cells from the noxious effects of the oxidizing agent H_2_O_2_. Results revealed that compared with the WT strain, disabling of *mfd* sensitized sporulating cells to H_2_O_2_; in contrast, the ∆GO cells exhibited a higher resistance to the oxidizing agent (Fig. 4A). Notably, the ∆*mfd* ∆GO strain exhibited a significantly higher susceptibility to H_2_O_2_ than the WT and ∆*mfd* strains (Fig. 4A); in summary, the WT, ∆*mfd*, ∆GO and ∆GO ∆*mfd* strains exhibited lethal dose 90 s (LD_90s_) of, 61 ± 4, 41 ± 3.5, 73 ± 2.5 and 11 ± 2, respectively (Fig. 4B). In nutritionally stressed non-replicating *B. subtilis* cells, the GO system prevented stress-associated mutagenesis while Mfd played a promutagenic role^21,27–29^. In the present report, we found that in sporulating cells, the absence of Mfd or the GO system induced a significant increase in the spontaneous and H_2_O_2_-promoted mutagenesis in comparison with the WT strain (Fig. 5). Of note, during sporulation, the spontaneous and induced mutagenesis values were significantly higher in the ∆GO ∆*mfd* strain than in the Mfd- or ∆GO-deficient strains (Fig. 5). Altogether, these results suggest that Mfd and the GO system protect sporulating cells from ROS-promoted DNA lesions that compromise sporulation.

### Cytological analysis of strain *B. subtilis* WT and ∆GO ∆*mfd* by epifluorescence and TE-microscopy

**Figure 6:**
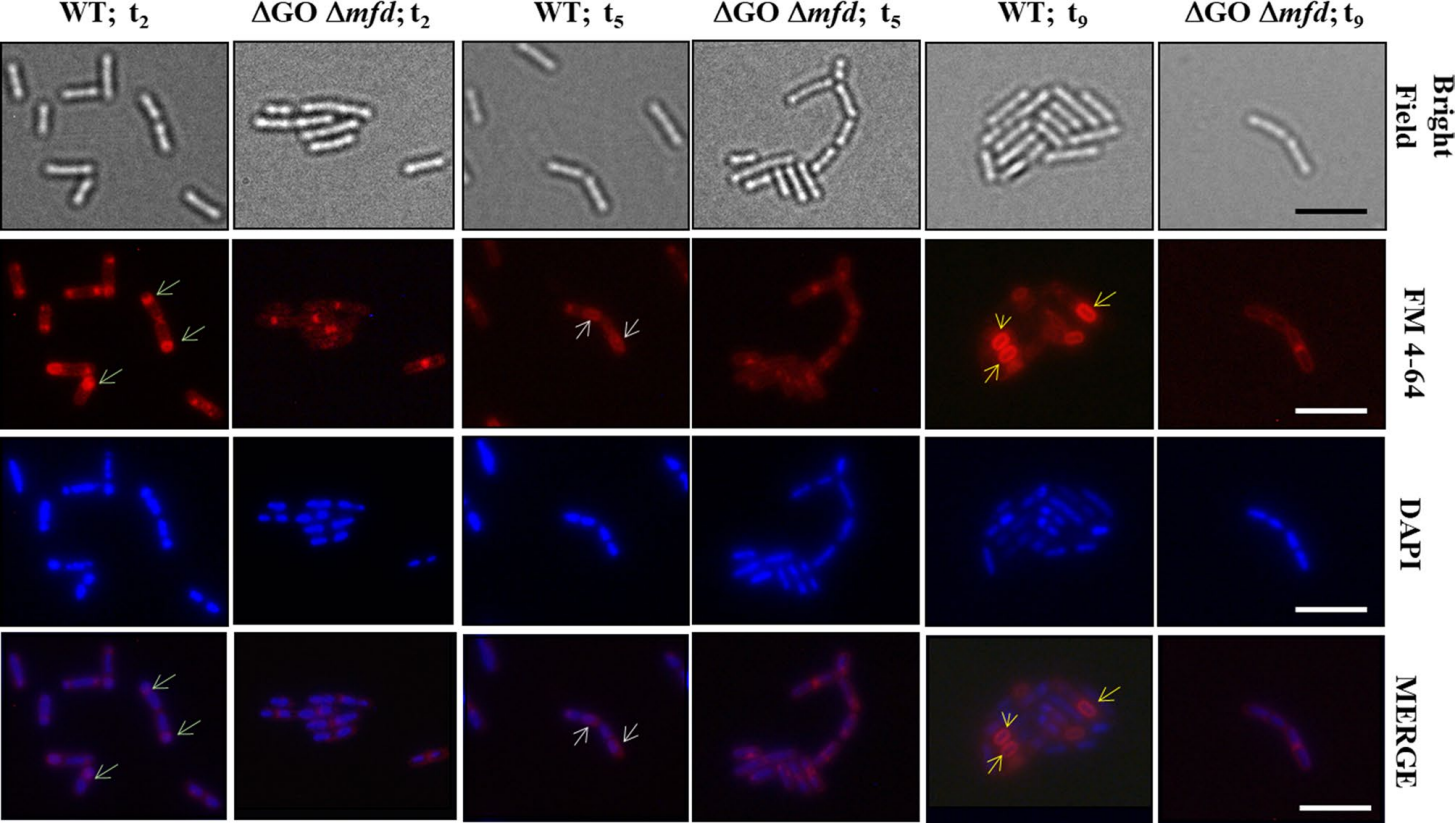
Epifluorescence microscopic analysis of sporulation stages of *B. subtilis* strains with a wild type (WT) or ∆*mfd*/∆GO genotype. The strains indicated were induced to sporulate in DSM. At the indicated stages (t_2_, t_5_ and t_9_), cells were collected, fixed and analyzed by bright- field (BF) and fluorescence (DAPI and FM4-64 staining) microscopy as described in “Materials and methods” section. Overlain images of DAPI and FM4-64 at each time point are depicted as MERGE. Yellow arrowheads show the proper development of the forespore in the WT strain during the stages analyzed. The scale bar is 5 μm and all images are at the same magnification.

As noted above, the simultaneous absence of a complete GO system and Mfd markedly decreased *B. subtilis* sporulation. Importantly, exponentially growing cells of this strain as well as those from strains with a disabled GO system or Mfd did exhibit similar doubling times and cellular morphologies as those detected in the wild-type strain by confocal microscopy (Fig. S1). In *B. subtilis* the sporulation process, which takes place during the stationary phase of growth, is defined by specific and well differentiated morphological steps (arbitrarily termed stages t_0_–t_9_)^1,3^. The first unequivocal manifestation of sporulation in *B. subtilis*, which occurs during stage t_2_, is characterized by the synthesis of an asymmetric cell division septum and the formation of a two-compartment asymmetric sporangium^3,30,31^. A subsequent temporal pattern of gene expression in each compartment drives the synthesis and maturation of an endospore^1,31–33^. Therefore, we examined ∆GO ∆*mfd* cells during sporulation for developmental morphological defects using epifluorescence (EF) and transmission electron microscopies (TEM). To this end, we collected samples of cultures at different stages during sporulation and stained their DNA and membrane with DAPI and FM4-64 dyes, respectively. In comparison to cells of the wild-type strain, the EF microscopic analysis revealed a number of morphological defects in the ∆GO ∆*mfd* mutant that began to manifest at the sporulation stage t_2_; firstly, the absence of cells with asymmetric septa (stage t_2_); secondly, the inability to progress into sporangia with well-defined mother cell and forespore compartments (t_5_), and, finally, the failure to generate mature endospores (t_9_) (Fig. 6).

**Figure 7:**
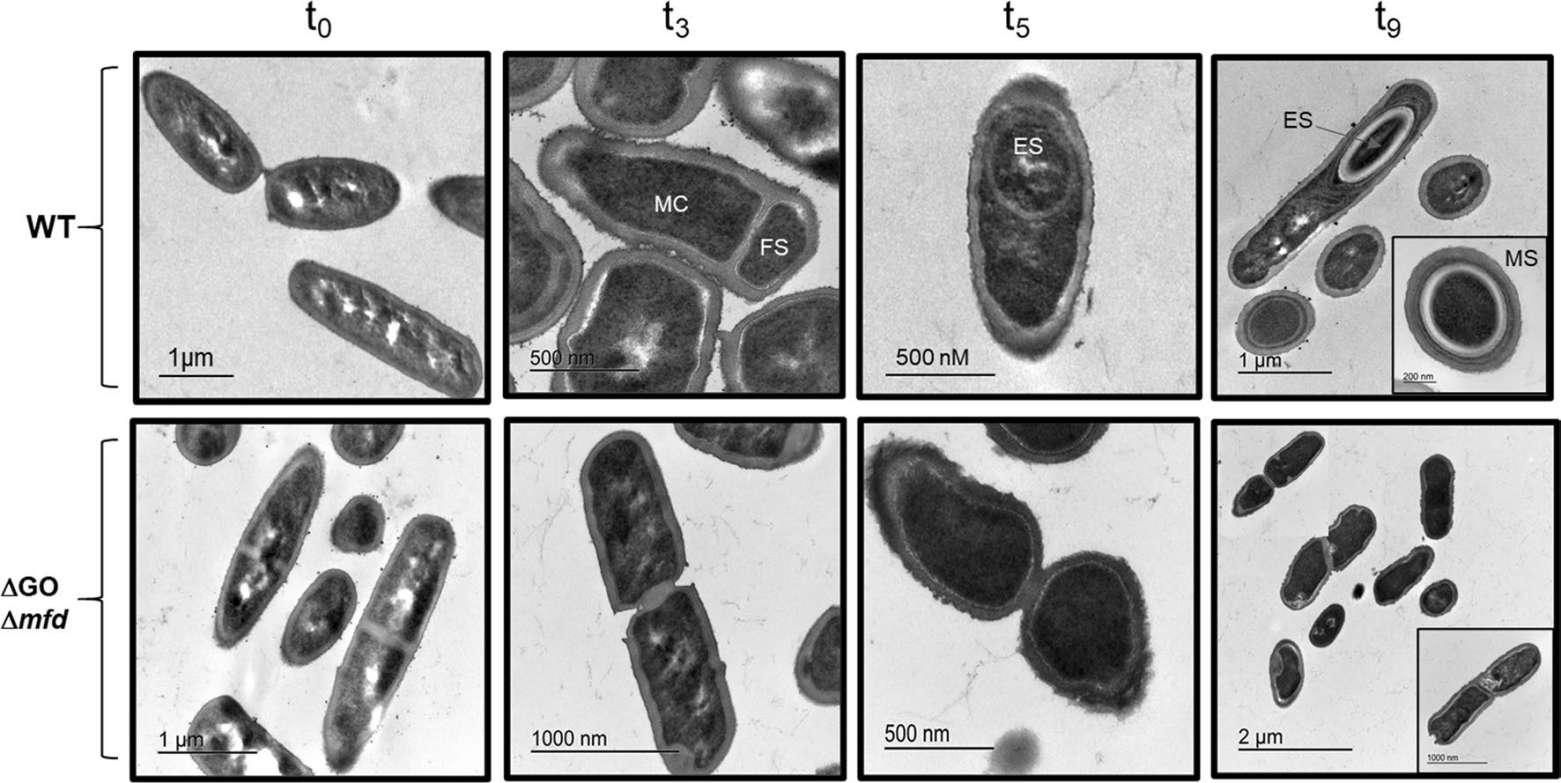
Transmission electron microscopy of sporulation stages of *B. subtilis* strains with a wild type (WT) or ∆*mfd* ∆GO genotype. The strains indicated were induced to sporulate in DSM. At the stages indicated (t_0_, t_3_, t_5_ and t_9_), cells were collected, fixed and analyzed by TEM as described in “Materials and methods” section. Red arrows indicate: *MC* mother cell, *FS* forespore, *ES* endospore, *MS* mature spore. Insets in stage t_9_ show: Upper panel, section of a mature spore; Lower pannel, section of an aberrant non-sporulated, divided cell. Scale bars as indicated in the panels.

The sporulation defect of the Mfd/GO-deficient strain, appeared to develop from the initial stages of sporulation and was analyzed in depth by TE microscopy. To this end, WT and ∆GO ∆*mfd* cells collected during sporulation stages t_0_, t_3_, t_5_ and t_9_ were fixed with glutaraldehyde and processed for TEM as described in Materials and Methods. During stage t_0_, both, the WT and the strain deficient for GO and Mfd, presented typical vegetative cellular morphologies; however, cells from the latter strain exhibited anomalous thick division septa (Fig. 7). Remarkably, the strain lacking the GO system and *mfd* did not experience the morphological events of asymmetrical cell division and forespore engulfment occurring during stages t_3_ and t_5_ in the wild type sporulating cells (Fig. 7). Indeed, during stages t_3_ and t_5_, the mutant generated dividing cells with aberrant morphologies and failed, during stage t_9_, to generate the typical sporangia with endospores and free spores as observed in the wild type strain (Fig. 7). In summary, these results not only attest for the failure in sporogenesis of the ∆GO ∆*mfd* strain, but also suggest that such phenotype was the product of defects that began at the initial stages of this developmental process.

### RecA, Sda, DisA and SirA regulate the sporulation defect of *B. subtilis* cells lacking a functional GO system and transcription-coupling repair

**Figure 8:**
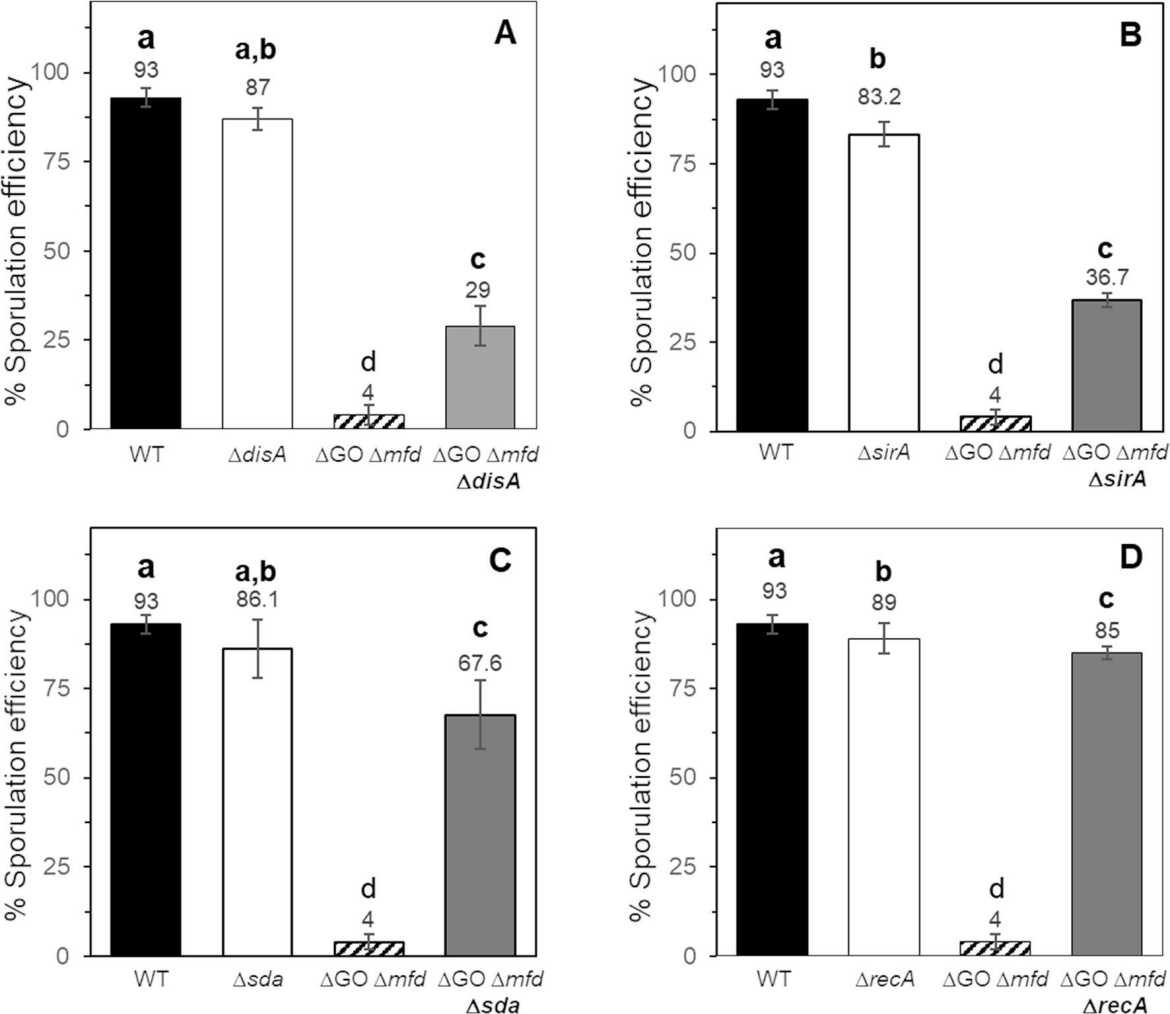
Analysis of suppressors of the sporulation defect of the strain *B. subtilis* ∆*mfd* ∆GO. The strains indicated were induced to sporulate in DSM for 24 h at 37 °C and assessed for sporulation efficiency by the heat-killing method. Letters a, b, c and d, indicate statistically significant differences between strains as determined by one-way analysis of variance (ANOVA) followed by a Tukey’s post-hoc test; *P* < 0.05. Values are expressed as mean values ± standard deviation of at at least three independent experiments.

The cellular defects exhibited by the ∆GO ∆*mfd* strain prompted us to investigate the existence of a possible checkpoint event blocking the onset of sporulation in this mutagenic, repair-deficient strain. Because the mutant failed to produce cells with typical morphologies in stage t_2_ sporangium, we first considered DisA. This octameric protein delays the segregation of one chromosomal copy to the sporangia’s forespore compartment when DNA damage is encountered during stages t_2_–t_3_^7,34^. The results of our genetic analysis revealed that disruption of *disA* significantly, but not completely, improved the sporulation efficiency of the quadruple knockout *ytkD mutM mutY mfd* strain (Fig. 8A). Based on these results we speculated that additional factor(s) must be involved in promoting the developmental defects observed in this mutant strain.

TEM analysis of the ∆GO ∆*mfd* strain showed a sporulation defect in this strain from the onset of sporulation (Fig. 7). Therefore, our analysis to identify suppressors of the sporulation defect exhibited by the ∆GO ∆*mfd* strain was extended to SirA (Sporulation inhibitor of Replication), a checkpoint protein that ensures the existence of a single chromosomal copy in each of the cell compartments of *B. subtilis* sporangia^8,35^. Disruption of *sirA* resulted in a partial restoration of the sporulation efficiency of the ∆GO ∆*mfd* strain following disruption of *sirA* (Fig. 8B). Therefore, it is possible to speculate that the exacerbated DNA damage occurring in the ∆GO ∆*mfd* strain activates SirA- and DisA-controlled checkpoint functions^7,8,34,35^. It has been shown that sporulation is inhibited if cells committed to this developmental process sense genetic alterations or conditions that interfere with DNA replication^6,8,16^, and that this inhibition is mediated by the multifunctional protein RecA^9,15^. Strikingly, the levels of sporulation efficiency were almost completely reestablished to those exhibited by the wild-type strain following disruption of *recA* in the ∆GO ∆*mfd* strain (Fig. 8D). In response to replicative stress and activation of the SOS-response, stationary phase cells induce the synthesis of SdA, a protein that specifically inhibits KinA’s autokinase activity, which results in reduced levels of Spo0A-P^6,36^. Our assays revealed that the genetic disruption of *sda* restored the sporulation efficiency of the strain lacking Mfd and a functional GO system (Fig. 8C). Taken together, these results strongly suggest that the DNA-damage dependent checkpoint proteins RecA, Sda, SirA and DisA, in a hierarchical manner (i.e., RecA > Sda > SirA > DisA) regulate the sporulation defect of a *B. subtilis* strain deficient in transcription coupling repair and prone to accumulate 8-OxoG lesions.

The major effect of RecA and Sda in suppressing the sporulation defect of the ∆GO ∆*mfd* strain prompted us to investigate whether its coding genes are upregulated in this genetic background. To test this notion, *recA-lacZ* and *sda-lacZ* transcriptional fusions were recombined in the *recA* or *sda* loci of the WT and ∆GO ∆*mfd* genetic backgrounds and the levels of β-galactosidase were determined in the resulting strains during the sporulation stage t_0_. The levels of β-galactosidase exhibited by the strain carrying the *recA*-*lacZ* fusion were ~ 3.9 times higher in the GO/Mfd-deficient strain than in the WT strain (i.e., 18.2 ± 0.7 vs 4.7 ± 0.7 Miller units). Furthermore, in the strains harboring the *sda-lacZ* fusion the levels of the reporter *lacZ* gene were ~ 11.9 times higher in the ∆GO ∆*mfd* strain than in the WT strain (i.e., 11.9 ± 1.1 vs 0.9 ± 0.1 Miller units). Altogether, these results strongly suggest that the levels of *recA* and *sda* are upregulated in the strain deficient in Mfd and the prevention/repair GO system.

## Discussion

Here, we report that the absence of the TCR factor Mfd, induced a marked decrease in the sporogenesis of a *B. subtilis* strain deficient for the repair/prevention GO system. As revealed by TEM and EF microscopies, this mutant failed to generate typical sporangia and developing mature spores. Also, it was found that RecA, Sda, SirA and DisA, which regulate sporulation checkpoint events, are responsible of the sporulation defects observed in the GO/Mfd-deficient strain.

In addition to its classical role in transcriptional coupling repair^14,19^, alternative functions have been attributed to Mfd in *B. subtilis*; namely, in conferring protection against protein oxidation^37^, as a regulator of carbon catabolite repression as well as in transcription associated mutagenesis of amino acid starved cells^27,28,38^.

Here and a previous report^13^ revealed an unexpected role for Mfd in endospore formation; essentially, in the absence of external DNA damaging factors, disruption of *mfd* resulted in a significant decrease in the efficiency of *B. subtilis* to generate spores. This result strongly suggests that spontaneous genetic lesions, other than those that cause major DNA distortions, interfere with the expression of genes that are necessary for an efficient sporogenesis. Two lines of evidence support this notion, firstly, around two hundred genes involved in sporulation were repressed during the stationary phase of growth in a Mfd-deficient strain^39^. Secondly, genetic disabling of BER-encoding genes that process AP sites and the highly mutagenic lesion 8-OxoG exacerbated the sporulation defect of the strain lacking Mfd (Figs. 1, 2). Importantly, disabling of all the components of the GO system resulted in a marked decline in sporogenesis in the strain deficient for TCR suggesting multiple types of genetic damages, including, (i) direct oxidation of guanines in DNA, (ii) accumulation of 8-OxG:A and G:A mispairs, and, (iii) oxidation of deoxy-GTP and GTP pools can directly or indirectly contribute to the sporulation defect observed in the Mfd/GO-deficient strain. Therefore, in addition to its TCR-NER functions^13^, here, we demonstrated that, under conditions of sporulation, Mfd together with the GO system, confers protection to *B. subtilis* cells from the cytotoxic and mutagenic effect of the ROS promoter agent H_2_O_2_. Altogether, these results suggest that when ROS-promoted DNA damage is encountered by stationary-phase cells committed to sporulation, Mfd elicits high-fidelity repair events and prevents mutagenesis. Of note, our sporulation results contrast those observed in non-growing stationary-phase *B. subtilis* starved cells; Mfd promoted mutagenesis genetic diversity that increased the likelihood of scaping growth-limiting conditions^27,28,40^.

Several lines of research have shown that DNA replication is intimately coordinated with the initiation of sporulation in *B. subtilis*; in this interplay, Sda, SirA, DisA and RecA play prominent roles^6–8,41,42^. Accordingly, results from ultrastructural, confocal and epifluorescence microscopies showed that the absence of Mfd and the GO system generated aberrant cells that failed to develop typical sporangia and progress to advanced sporulation stages (Figs. 6, 7, S2). Furthermore, our suppressors analysis revealed that disruption of *disA*, *sirA*, *recA* and *sda*, restored to different levels the ability to sporulate of the quadruple *mutM mutY ytkD mfd* strain. Previous results have revealed that transcription of *sirA* is activated at the start of sporulation, under control of the master regulator Spo0A, to inhibit replication to the existence of only two chromosomal copies in cells committed to sporulation^8,33,35^. Inactivation of *sirA* partially relieved the sporulation efficiency in the strain deficient for Mfd and the GO system. Therefore, in addition to interfering with replication, SirA seems to play additional roles in sporulation, as cells of this TCR/repair deficient strain were uncapable of adopting typical morphologies of sporulating cells (Fig. 7). Our results also revealed a partial suppression of the sporulation defect exhibited by the GO/Mfd-deficient strain in a DisA-deficient background. We hypothesize that, during the stage *t*_2_ of sporulation, a subpopulation of cells of the quadruple *mfd mutM mutY ytkD* mutant strain accumulate DNA lesions that elicit a DisA-dependent checkpoint event that aborts the establishment of the two cell type sporangia. In support of this notion, (i) during sporulation, the strain deficient for GO and Mfd exhibited repair deficiencies and increased mutagenesis under conditions of oxidative stress, and, (ii) as evidenced by TEM, this mutant did not establish asymmetrically divided sporangia. In *B. subtilis* proficient for Mfd and the GO system, the RecA-dependent SOS response is active and required to protect sporulating cells from the DNA damaging factors UV-C light and M-C^9^. Our results revealed that the marked decrease in the sporulation efficiency observed in the strain deficient for Mfd and the GO system is regulated by RecA; disruption of its encoding gene restored the sporulation efficiency of this mutant to levels slightly lower levels than those exhibited by the WT strain. Based on these observations, is feasible to speculate that in starved, Mfd/GO-deficient *B. subtilis* cells, the accumulation of 8-OxoGs^29,43^ or its repair intermediates generates replication and/or transcription stress and activates the RecA-dependent SOS response. In support of this assertion, as shown in this work, the levels of *recA* and *sda* were found to be upregulated in the ∆GO ∆*mfd* mutant; furthermore, in the Sda^-^ background; Sda^−^ cells displayed an increase in sporulation efficiency of the GO/Mfd-deficient strain. Accordingly, it has been shown that in cells committed to sporulation the SOS-induction of *sda* inhibits phosphorylation of Spo0A and the initiation of this developmental pathway^6,36^. On the other hand, accumulation of unphosphorylated Spo0A activates the expression of *sirA*, the inhibitor of the replication initiator protein DnaA^35^. In addition, the DNA-damaging conditions prevailing in the ∆*mfd* ∆GO genetic background may converge in the generation of recombination intermediates that pause the scanning activity of DisA and interferes with the proper establishment of functional sporangia^7,44^.

RecA-dependent mechanisms that regulates the initiation of sporulation in *B. subtilis* has been previously described^17^. However, our results contribute two novel aspects to RecA-dependent regulation of the initiation of sporulation, (i) the ROS promoted lesion 8-OxoG can signal the activation of the function of RecA, and, (ii) Mfd couples the level of 8-OxoGs and transcription/replication stress to the activation of the RecA-dependent pathway to impact in the initiation of sporulation.

## Materials and methods

### Bacterial strains and growth conditions

The *B. subtilis* strains used in this study were derived from strain 168 and are listed in Table S1. The strains were constructed using standard molecular biology techniques^45^. *B. subtilis* strains were routinely incubated at 37 °C in Lysogeny–Broth (LB)^46^ medium or Difco sporulation medium (DSM)^47^, with shaking. When required, erythromycin (Ery; 5 μg/ml), chloramphenicol (Cm; 5 μg/ml), neomycin (Neo; 10 μg/ml), tetracycline (Tet; 15 μg/ml), spectinomycin (Spc; 100 μg/ml), or rifampicin (Rif; 10 μg/ml) were added to media. For culture of *E. coli* strains harboring plasmids, 100 μg/ml ampicillin was added to the LB medium. Solid media were obtained by adding bacteriology grade agar (15 g/l) to the liquid media.

Transformation of *B. subtilis* was performed through natural competence^48^. Detailed description of *B. subtilis* strains is presented in Supplementary material.

### Strains construction

A gene construct to disrupt *sirA* was generated as follows. A 234‐bp DNA fragment extending from nucleotides (nt) 58–291 from the *sirA* open reading frame (ORF) was PCR amplified using chromosomal DNA from *B. subtilis* 168. The oligonucleotide primers used for this reaction were 5′-CGGAATTCGGCCGGGAATCGGTTATGTTTGAG-3′ (forward) and 5′-GCGGATCCCTTCATCATAAACGTCGCGTG-3′ (reverse). Restriction sites (underlined) were included in the primers for cloning the amplified product between the EcoRI-BamHI and cloned into the pMutin4cat vector^49^ using the *E. coli* strain DH5α. The resulting plasmid pPERM1791 was used to transform *B. subtilis* 168 and PERM1136 to generate *B. subtilis* strains ∆*sirA* (PERM1796) and ∆*ytkD* ∆*mutM* ∆*mutY* ∆*sirA* (PERM1798). To generate a *B. subtilis* strain deficient for GO, Mfd and SirA, competent cells of the strain PERM1390 (Table S1) were transformed with plasmid pPERM1791 to generate the strain *B. subtilis* PERM1801 (ΔGO Δ*mfd* Δ*sirA*)*.*

The ΔGO Δ*sda* Δ*mfd* mutant in the 168 background was generated by transforming strain PERM1808 (Δ*sda*) with genomic DNA isolated from *B. subtilis* strain PERM1573 (ΔGO) (Table S1), to generate the strain PERM1815 (ΔGO Δ*sda*). Subsequently, the genetic inactivation of *mfd* was achieved by transforming competent cells of *B. subtilis* strain PERM1815 with the plasmid pPERM1538 (Cm^R^) (Supplemental Table S1), thus generating the strain *B. subtilis* PERM1818 (ΔGO Δ*sda* Δ*mfd*)*.*

A *B. subtilis* strain deficient for GO, RecA and Mfd was constructed as follows. To disrupt *mfd*, competent cells of *B. subtilis* ∆GO (PERM1136)^26^ were independently transformed with plasmid pPERM1291 (Table S1) and chromosomal DNA from strain *B. subtilis* PERM688 (Δ*recA*), generating strains *B. subtilis* ∆GO ∆*mfd* (PERM1390) and ∆GO ∆*recA* (PERM1740), respectively. Subsequently, competent cells of the strain PERM1740, were transformed with the plasmid pPERM1291 (Table S1) leading to disruption of *mfd*; thus, generating strain *B. subtilis* PERM1745 (ΔGO Δ*recA* ∆*mfd)*. Disruption of *disA* in the genetic background *B. subtilis* PERM1390, was achieved by transforming competent cells of this strain with plasmid pPERM1372^14^_,_ to generate the strain *B. subtilis* PERM1751 (ΔGO Δ*recA* ∆*disA*). The appropriate recombination events into the homologous loci were confirmed by PCR using specific oligonucleotide primers and by antibiotic resistance.

The construction of transcriptional *sda*-*lacZ* and *sirA*-*lacZ* fusion was performed with the integrative vector pMutin4-cat^49^. To this end, a 180-bp internal fragment of the *sda* gene was amplified by PCR with Vent DNA polymerase (New England BioLabs) using chromosomal DNA from *B. subtilis* 168. The oligonucleotide primers used for the amplification of *sda* fragment were 5′-GCGAATTCCAACTTTTAAGGAGGTGCC-3′ (forward) and 5′-GCGGATCCTACGGAAATAATATGTCCGAGCGA-3′ (reverse). The *sda* PCR fragment was ligated between the EcoRI and BamHI sites of pMUTIN4-cat. The resulting construct, designated pPERM1790 (*sda*::*lacZ*) was propagated in *E. coli* DH5α cells. Plasmid pPERM1790 was used to transform *B. subtilis* 168 and PERM1390 (∆GO ∆*mfd*), generating strains *B. subtilis* PERM1797 (*sda*-*lacZ*) and PERM1802 (∆GO ∆*mfd sda*-*lacZ*), respectively (Table S1). To generate a strain carrying a *recA*-*lacZ* fusion in the WT and ∆GO ∆*mfd* genetic background competent cells of strain *B. subtilis* 168 and PERM1390 (∆GO ∆*mfd*) were transformed with chromosomal DNA isolated from *B. subtilis* PERM115^13^, thus generating the strain *B. subtilis* PERM1824 and PERM1825, respectively (Table S1).

### Sporulation assays

To determine sporulation efficiency, strains were assessed for the presence of heat-resistant spores, as previously described^13^. Briefly, cells were grown in liquid Difco Sporulation Medium (DSM) for 24 h at 37 °C with shaking. At this time, the total viable CFU/ml was measured by plating aliquots of serial dilutions in PBS and then the cultures were heated at 80 °C for 20 min; the viability was assessed again to determine the number of spores present in each sample.

### β-galactosidase activity assay

*B. subtilis* strains 1797, 1802, 1824 and 1825 (Table S1) were grown and sporulated in liquid DSM at 37 °C. Cells samples (1 ml) were harvested by centrifugation during sporulation stage t_0_ and the pellets were washed twice with 50 mM Tris–HCl (pH 7.5) and stored at − 20 °C. Cells were disrupted with lysozyme followed by centrifugation, and the levels of β-galactosidase was determined according to a previously described protocol^50^, using *ortho*-nitrophenyl-β-d-galactopyranoside (ONPG) as the substrate, and the β-galactosidase activity was expressed in Miller units^46^.

### Electron microscopy

For transmission electron microscopy analysis, 10 ml samples of NSM cultures from *B. subtilis* strains WT and PERM1390 (∆GO ∆*mfd*), collected during sporulation stages (t_0_, t_3_, t_5_, and t_9_), were mixed with 10 ml of 4% (W/V) glutaraldehyde in 0.1 M sodium Cacodylate buffer, pH 8.3 (Fixation buffer; FB) and incubated 30 min at room temperature. The cell suspension was centrifuged (3800×*g*) for 10 min at room temperature; after eliminating the supernatant, the cell pellets were resuspended in 5 ml of FB and incubated overnight at 4 °C. Next day, the cell samples were washed 5 times with glutaraldehyde-free FB and resuspended in 25 ml of 0.1% (V/V) OsO_4_ in 0.05 M sodium cacodylate buffer, pH 7.5 and incubated 24 h at 4 °C. After pelleting (3800×*g*; 12 min/4 °C), the cells were washed 3 times with 0.1 M sodium Cacodylate buffer, pH 7.5 and three times with MQ water. The cell samples pelleted by centrifugation as indicated above, were resuspended in 1% (W/V) melted agar and the suspension was settled at room temperature until solidifying. The agar blocks were cutted in ~ 2 mm slices, mixed with 3% (V/V) uranyl acetate and stored overnight at 4 °C. After adjusting at room temperature, the agar slices were washed 3 times, for 30 min, with MQ water and stored at 4 °C^51^.

Agar embedded cells were then dehydrated through an ethanol series. Next, samples were immersed in propylene oxide and embedded in Epon 812 resin (Electron Microscopy Sciences, Inc., Hatfield PA) and polymerized at 60 °C for 48 h. Sectioned on a Leica Ultracut R ultramicrotome (Leyca Microsystems, Wetzlar, DEU), and stained with 4% uranyl acetate and 0.3% Reynold lead citrate^49^. Micrographs were taken on a Jeol JEM-1010 (Jeol, Inc., JPN) electron microscope with an accelerating voltage of 80 kV and electron micrographs were captured with a CCD camera model Gatan Orius SC600 and a digital micrograph software.

### Treatment of sporulating cells with the oxidizing agent H_2_O_2_

Strains were propagated in DSM under vigorous aeration (250 rpm) at 37 °C and their growth was monitored by optical density at 600 nm (OD_600_). At 4.5 h after cessation of exponential growth (t_4.5_), the culture was exposed to different doses of H_2_O_2_ in a concentration range of 0 to 75 mM for additional 1 h. The survival of sporulating cells during this treatment was measured by plating aliquots of serial dilutions in PBS on LB medium agar plates. The plates were grown overnight at 37 °C and the viable count was performed to determine the lethal doses 50 (LD_50_) and 90 (LD_90_).

### Determination of spontaneous and H_2_O_2_-induced mutation frequencies in sporulating cells

Spontaneous and hydrogen peroxide induced Rif^R^ mutagenesis was performed according to a previously described protocol^13^. Briefly, strains were induced to sporulate at 37 °C in DSM. 4.5 h after t_0_ (the time when the exponential and stationary phase slots intersect), the cultures were equally divided in two subcultures. One of these subcultures was left untreated and the other was amended with a lethal dose fifty (LD_50_) of H_2_O_2_. Mutation frequencies to Rif^r^ were determined by plating aliquots of cells on six LB plates containing 10 mg/ml of rifampicin. Colonies resistant to rifampicin were counted after 24 h. The number of cells to calculate the mutation frequencies were obtained from aliquots of appropriate dilutions of the cultures plated on solid LB medium lacking rifampicin that were incubated for 24 h at 37 °C. The experiment was repeated at least three times.

### Fluorescence microscopy

Fluorescence microscopy analysis of cells during sporulation was determined as previously described^9,13,34^. Briefly, *B. subtilis* strains wild-type and ΔGO Δ*mfd* were propagated in DSM at 37 °C. Cellular samples of both cultures were collected during 2, 5 and 9 h after t_0_, corresponding to sporulation stages t_2_, t_5_ and t_9_, respectively. Cell samples collected at the appropriate times were fixed, stained with DAPI and FM4-64 as previously described^34^ and analyzed by epifluorescence microscopy employing a Zeiss Axioscope A1 microscope equipped with an AxioCam ICc1 camera. Fluorescence and bright field images were acquired by using AxioVision V 4.8.2 software and adjusted only for brightness and contrast as previously described^9,13,34^. Conditions employed for excitation and emission wavelengths were 350 and 470 nm for DAPI and 506 and 750 nm for FM4-64, respectively^9,13,34^.

### Confocal microscopy

Cell morphology during growth and sporulation was analyzed by phase contrast using confocal microscopy. *B. subtilis* cells were grown and induced to sporulate in DSM. Cellular samples of the wild-type strain, ΔGO Δ*mfd*, ΔGO and Δ*mfd* mutants were collected at the appropriate times, washed twice with cold phosphate-buffered saline [PBS; 0.7% Na2HPO4, 0.3% KH2PO4, 0.4% NaCl (pH 7.5)] and were fixed as described previously^20^. Phase contrast microscopy was performed with a Zeiss LSM700 scanning laser confocal microscope with a LD A-Plan 40x/0.55 Ph2 objective. Images were acquired and adjusted only for brightness and contrast with image software (Zen 2011, Carl Zeiss MicroImaging GHBH, Jena, Germany).

### Statistical methods

For determination of sporulation frequency, lethal doses 90 (LD_90_) and mutagenesis rate differences were calculated by performing one‐way Analysis of Variance (ANOVA) followed by a Tukey’s post‐hoc analysis. Significance was set a *P* < 0.05. Analyses were performed with the OriginPro 2017.

## Supplementary Information

Supplementary Information.
