# Supplementary material for: Transcriptional coupling and repair of 8-OxoG activate a RecA-dependent checkpoint that controls the onset of sporulation in Bacillus subtilis
Source: Sci Rep. 2021 Jan 28;11:2513. doi: 10.1038/s41598-021-82247-8 (PMC7844254; doi:10.1038/s41598-021-82247-8)
Supplement: Supplementary file 1 — Supplementary Information. [file 41598_2021_82247_MOESM1_ESM.pdf]

SUPPLEMENTARY INFORMATION

Transcriptional Coupling and Repair of 8-OxoG Activate a RecA-Dependent Checkpoint that Controls the Onset of Sporulation in *Bacillus subtilis*

Valeria P. Suárez[a&], Lissett E. Martínez[a&], Hilda C. Leyva-Sánchez[a], Luz I. Valenzuela-García[a], Reyna Lara-Martínez[c], Luis F. Jiménez-García[c] Norma Ramírez-Ramírez[a], Armando Obregon-Herrera[a], Mayra Cuellar-Cruz[a], Eduardo A. Robleto[b], Mario Pedraza-Reyes[a#].

Department of Biology, Division of Natural and Exact Sciences, University of Guanajuato, Guanajuato, México[a]; School of Life Sciences, University of Nevada, Las Vegas, Nevada, USA[b]; Department of Cell Biology, Faculty of Sciences, National Autonomous University of Mexico (UNAM), Circuito Exterior, Ciudad Universitaria, Cd. Mx., Coyoacán, 04510 Mexico City, Mexico[c].

Running Head: Mfd and 8-OxoG regulation of *B. subtilis* sporulation

# Address correspondence to Mario Pedraza-Reyes, pedrama@ugto.mx

[&] These authors contributed equally to this work

SUPPLEMENTAL LEGEND S1-S2

FIG S1. Growth kinetics (OD_{600nm}), doubling times, and morphologies of *B. subtilis* strain 168 derivatives. The strains *B. subtilis* wild type (**A**), ΔGO (**B**), Δ*mfd* (C) and ΔGO Δ*mfd* (**D**) were propagated in DSM. At the indicated times, culture samples of each strain were collected to determine OD_{600nm}. Insets show cells from cultures collected at an OD_{600nm} = 0.75, were analyzed by confocal microscopy as described in Materials and Methods. **dt**, doubling times. The scale bar is 2 μm.

FIG S2. Confocal microscopic analysis of sporulation stages of *B. subtilis* strains with a wild type (WT) or Δ*mfd*/ΔGO genotype. The strains indicated were induced to sporulate in DSM. At the indicated stages (t_0, t_5, t_9 and t_{20}), cells were collected and analyzed by confocal microscopy as described in Materials and Methods. The scale bar is 2 μm.

SUPPLEMENTAL FIGURE S1

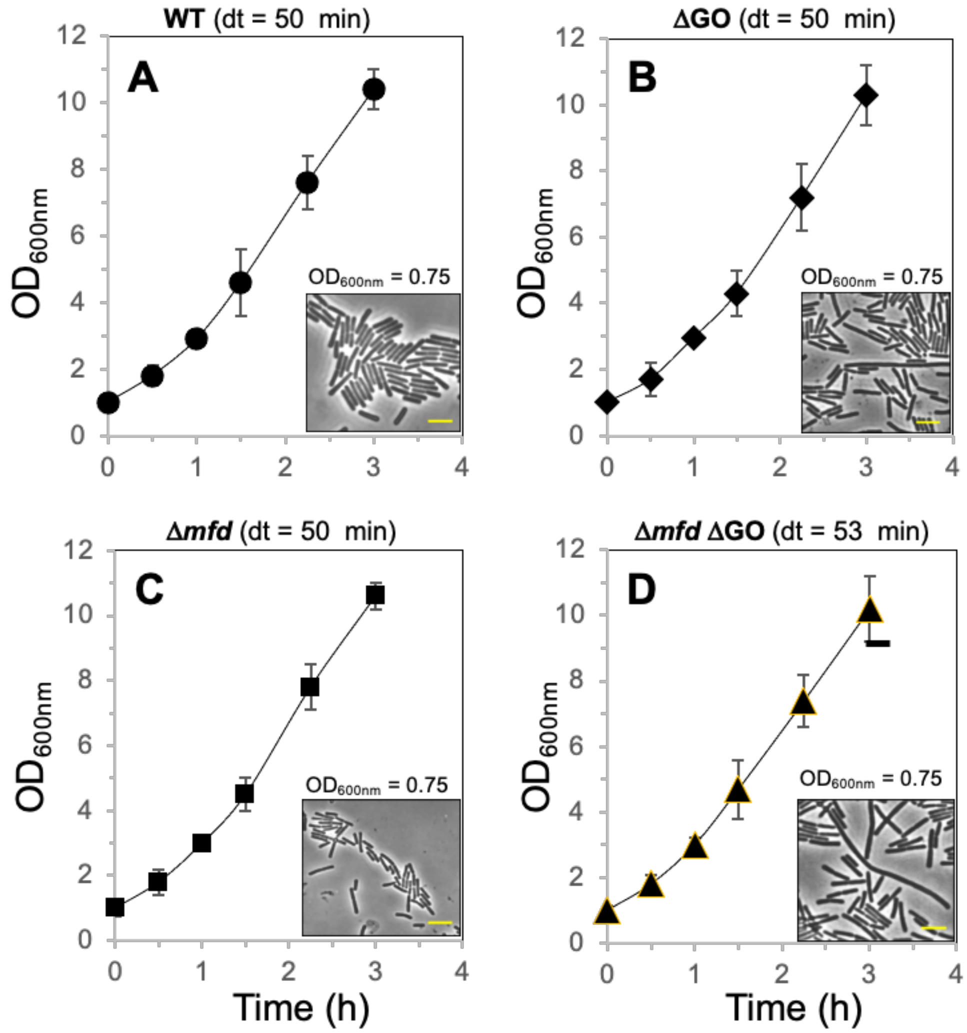

SUPPLEMENTAL FIGURE S2

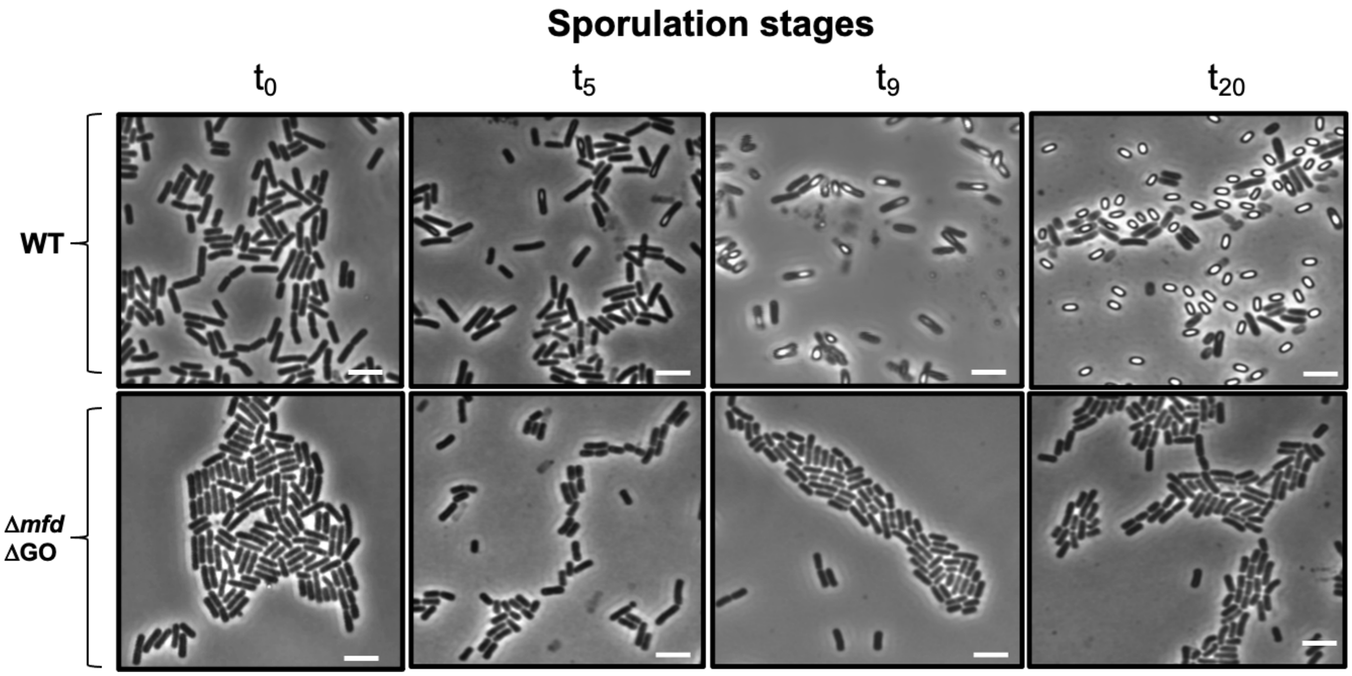

SUPPLEMENTAL TABLE S1.

Table S1. Strains and plasmids used in this study

Strain or plasmid	Genotype and/or relevant features[a]	Source, reference, or construction[b]
E. coli		
DH5α	F⁻ φ80*lac*ZΔM15 Δ(*lac*ZYA-*arg*F) U169 *rec*A1 *end*A1 *hsd*R17(r_K^- ,m_K^+) *pho*A *sup*E44 λ⁻ *thi*-1 *gyr*A96 *rel*A1	Invitrogen
B. subtilis		
168	Wild type, *trp*C2	Laboratory stock
PERM595	Δ*ytkD::neo* Neo^r	Castellanos Juárez FX. *et al.,* 2006
PERM688	Δ*recA*::*cat* Cm^r	Sung HM and Yasbin RE. 2002
PERM1136	ΔGO (*ytkD::neo mutM::tet mutY::ery*) Neo^r Tet^r Ery^r	Vidales LE. *et al.,* 2009
PERM1307	ΔAP (*nfo::neo exoA::tet nth::ery*) Neo^r Tet^r Ery^r	Barajas-Ornelas *et al*., 2014
PERM1309	Δ*mfd::spc* Spc^r	pPERM1291→PERM168
PERM1310	ΔAP (*nfo::neo exoA::tet nth::ery*) Δ*mfd::spc* Neo^r Tet^r Ery^r Spc^r	pPERM1291→PERM1307
PERM1390	ΔGO*::*Neo^r Tc^r Ery^r Δ*mfd::*spc Neo^r Tc^r Ery^r Spc^r	pPERM1291→PERM1136
PERM1504	Δ*disA::cat* Cm^r	pPERM1372→168
PERM1573	ΔGO (*ytkD::neo mutM::tet mutY:: spc*) Neo^r Tet^r Spc^r	Laboratory stock
PERM1701	Δ*mutM::cat* Cm^r	Gómez-Marroquín M. *et al.,* 2015
PERM1702	Δ*mutY::ery* Ery^r	pPERM979→PERM168
PERM1712	Δ*mutM::cat* Δ*mutY::ery* Cm^r Ery^r	pPERM979→PERM1701
PERM1713	Δ*mutM*::*cat* Δ*ytkD::neo* Cm^r Neo^r	PERM1701→PERM498
PERM1714	Δ*mutY*::*ery* Δ*ytkD::neo* Ery^r Neo^r	pPERM979→PERM498

PERM1731	Δ*mutM*::*cat* Δ*mfd::*spc Cm^r Spc^r	pPERM1291→PERM1701
PERM1732	Δ*mutY::ery* Δ*mfd::spc* Ery^r Spc^r	pPERM1291→PERM1702
PERM1733	Δ*ytkD::neo* Δ*mfd::spc* Neo^r Spc^r	pPERM1291→PERM498
PERM1734	Δ*mutM::cat* Δ*mutY::ery* Δ*mfd::spc* Cm^r Ery^r Spc^r	pPERM1291→PERM1712
PERM1735	Δ*mutM::cat* Δ*ytkD::neo* Δ*mfd::spc* Cm^r Neo^r Spc^r	pPERM1291→PERM1713
PERM1736	Δ*mutY::ery* Δ*ytkD::neo* Δ*mfd::spc* Ery^r Neo^r Spc^r	pPERM1291→PERM1714
PERM1737	ΔGO*::*Neo^r Tc^r Ery^r Δ*disA::cat* Neo^r Tc^r Ery^r Cm^r	pPERM1372→PERM1136
PERM1740	ΔGO*::*Neo^r Tc^r Ery^r Δ*recA::cat* Neo^r Tc^r Ery^r Cm^r	PERM688→PERM1136
PERM1751	ΔGO*::*Neo^r Tc^r Ery^r Δ*mfd::spc* Δ*disA::cat* Neo^r Tc^r Ery^r Spc^r Cm^r	pPERM1372→PERM1390
PERM1745	ΔGO*::*Neo^r Tc^r Ery^r Δ*mfd::spc* Δ*recA::cat* Neo^r Tc^r Ery^r Spc^r Cm^r	pPERM1291→PERM1740
PERM1796	Δ*sirA::cat* Cm^r	pPERM1791→PERM168
PERM1797	*sda-lacZ* Cm^r	This study
PERM1798	ΔGO*::*Neo^r Tc^r Ery^r Δ*sirA::cat* Neo^r Tc^r Ery^r Cm^r	pPERM1791→PERM1136
PERM1801	ΔGO*::*Neo^r Tc^r Ery^r Δ*mfd::*spc Δ*sirA::cat* Neo^r Tc^r Ery^r Spc^r Cm^r	pPERM1791→PERM1390
PERM1802	ΔGO*::*Neo^r Tc^r Ery^r Δ*mfd::*spc *sda-lacZ* Cm^r	This study
PERM1808	Δ*sda::ery* Ery^r	BGSC
PERM1809	Δ*sda::cat* Cm^r	BGSC
PERM1815	ΔGO*::*Neo^r Tc^r Spc^r Δ*sda::ery* Neo^r Tc^r Spc^r Ery^r	This study
PERM1818	ΔGO*::*Neo^r Tc^r Ery^r Δ*sda::ery* Δ*mfd::cat* Neo^r Tc^r Ery^r Spc^r Cm^r	This study
PERM1824	*recA-lacZ* Cm^r	This study
PERM1825	ΔGO*::*Neo^r Tc^r Ery^r Δ*mfd::*spc *recA-lacZ* Cm^r	This study
**Plasmids**		

pMutin4-cat	Integrational *lacZ* fusion vector; Cm^r	Barajas-Ornelas *et al*., 2014
pPERM979	pMUTIN4 containing an internal region (386 bp) of *mutY;* Em^r	Gómez-Marroquín M, *et al*., 2016
pPERM1291	pJET1.2/blunt containing the spectinomycin resistance cassette inside the *mfd* ORF*;* Spc^r	Laboratory stock
pPERM1372	pMutin4cat containing an internal region (307 bp) of *disA*; Cm^r	Valenzuela-García LI, *et al*., 2018
pPERM1538	pJET1.2/blunt containing the chloramphenicol resistance cassette inside the *mfd* ORF*;* Cm^r	Laboratory stock
pPERM1790	pMutin4cat containing the RBS site and a fragment of the *sda* 180 bp gene cloned with the EcoRI and BamHI sites; Cm^r	This study
pPERM1791	pMutin4cat containing an internal region (294 bp) of *sirA*; Cm^r	This study

[a] Selection marker: Amp, ampicillin; Cm, chloramphenicol; Kan, kanamycin; Spc, spectinomycin; Tc, tetracycline; Ery, erythromycin.

[b] X→Y Indicates that the strain Y was transformed with DNA from source X.

[C] BGSC: *Bacillus* genetic stock center.
